# Increased Risk of Acute Coronary Syndrome in Ankylosing Spondylitis Patients With Uveitis: A Population-Based Cohort Study

**DOI:** 10.3389/fimmu.2022.890543

**Published:** 2022-06-10

**Authors:** Kathy Ming Feng, Wu-Chien Chien, Yi-Hao Chen, Chien-An Sun, Chi-Hsiang Chung, Jiann-Torng Chen, Ching-Long Chen

**Affiliations:** ^1^ Department of Ophthalmology, Tri-Service General Hospital, National Defense Medical Center, Taipei, Taiwan; ^2^ Department of Medical Research, Tri-Service General Hospital, National Defense Medical Center, Taipei, Taiwan; ^3^ School of Public Health, National Defense Medical Center, Taipei, Taiwan; ^4^ Taiwanese Injury Prevention and Safety Promotion Association, Taipei, Taiwan; ^5^ Graduate Institute of Life Sciences, National Defense Medical Center, Taipei, Taiwan; ^6^ Department of Public Health, College of Medicine, Fu-Jen Catholic University, New Taipei City, Taiwan; ^7^ Big Data Research Center, College of Medicine, Fu-Jen Catholic University, New Taipei City, Taiwan

**Keywords:** acute coronary syndrome, ankylosing spondylitis, uveitis, cardiovascular disease, epidemiology

## Abstract

**Background:**

Uveitis, a sight-threatening ocular inflammatory state, is associated with autoimmune diseases and systemic inflammation. This prolonged systemic inflammation may cause plaque formation in coronary arteries, subsequently resulting in acute coronary syndrome (ACS).

**Methods:**

This retrospective, population-based study (15-year period) used the Longitudinal Health Insurance Database based on the National Health Insurance Research Database in Taiwan. Chi-square and Student’s t-tests were used to examine differences between the study and comparison cohorts for categorical and continuous variables, respectively. Fine and Gray’s competing risk model was used to determine the hazard ratio of the risk of ACS. Furthermore, the cumulative risk of ACS was determined using Kaplan-Meier analysis.

**Results:**

A total of 1,111 patients with AS and uveitis were enrolled in this study cohort, and 4,444 patients with AS without uveitis were enrolled in the comparison cohort. After adjustment for age, sex, and comorbidities, patients with AS and uveitis demonstrated an increased risk of ACS compared to those without uveitis (adjusted hazard ratio: 1.675, p<0.001). In addition, Kaplan-Meier analysis revealed that patients with AS and uveitis had a significantly higher risk of ACS than those without uveitis (p<0.001). Age, diabetes mellitus, hypertension, hyperlipidemia, chronic obstructive pulmonary disease, asthma, and systemic steroids were significant risk factors for ACS. Both anterior uveitis and posterior segment involvement were associated with an increased risk of ACS in patients with AS. All-cause mortality was higher in the uveitis group (9.81%) than in the non-uveitis group (8.10%) (p=0.015).

**Conclusion:**

Our analysis revealed that uveitis could potentially be a predictor of ACS in patients with AS. However, further prospective controlled studies are required to assess the association between uveitis and ACS in patients with AS.

## Introduction

Ankylosing spondylitis (AS) is a chronic inflammatory arthritis that involves the axial skeleton, belonging to a group of diseases known as spondyloarthritis (1). Usually, AS presents in the third decade of life and occurs more commonly in men (2, 3). An increased risk of cardiovascular morbidity and mortality has been recognized in patients with AS (4, 5). Several studies have reported a higher incidence of acute coronary syndrome (ACS) in patients with AS than in the general population (6, 7). However, the exact underlying mechanism remains unknown. The pathogenesis of atherosclerosis involves immune cell aggregation and deposition of cholesterol in arterial walls (8). Previous studies have shown that inflammatory cytokines, oxidative stress, and activated T-cells can cause endothelial dysfunction and endovascular injury (9–11). Prolonged inflammation may cause the plaque to become unstable with subsequent rupture and thrombus formation, occlusion of vessels, and finally ACS. Thus, immune-mediated inflammatory disorders may contribute to ACS events through systemic inflammation rather than through commonly known risk factors.

Uveitis, or inflammation of the uvea, is the most frequent extra-articular manifestation of AS, followed by psoriasis and inflammatory bowel disease (2). Pro-inflammatory cytokines and chemokines are known for their role in the pathogenesis of uveitis, and studies have demonstrated an increase in cytokines and chemokines not only in aqueous and vitreous samples but also in tears and serum samples from patients with uveitis (12, 13). Thus, uveitis may imply active systemic inflammation in patients with AS. In addition, a cross-sectional study in Norway reported an increased odds ratio for atherosclerosis and hypertension in patients with AS with a history of uveitis (14). As inflammation attracts cytokines and chemokines, which may affect further coronary vascular endothelial injury, uveitis may be considered a risk factor for ACS in patients with AS. However, no study has examined this association. Undoubtedly, the health and economic burden of ACS is high, and ACS has the greatest mortality and disability-adjusted life years worldwide (15, 16). Although ACS mainly occurs in elderly patients, the incidence of ACS in younger patients is increasing (17). The consequences of ACS in younger, active patients can be devastating; hence, it is essential to understand the relationship between patients with AS and uveitis and the risk of ACS.

Taking into account the above-mentioned considerations, we designed a retrospective matched-cohort study that used claims data from the National Health Insurance Research Database (NHIRD), which encompasses a comprehensive longitudinal medical record from almost the entire population of Taiwan (23 million), to evaluate the association between patients with AS and uveitis and the risk of ACS.

## Materials and Method

### Data Source

This study employed the Longitudinal Health Insurance Database (LHID) from the NHIRD to examine the association between uveitis in patients with AS and subsequent development of ACS in Taiwan over a 15-year period. The claims data contained medical records of outpatients and inpatients and were registered using the International Classification of Diseases, Ninth Revision, Clinical Modification (ICD-9-CM) codes. This NHIRD included around 99% of 23 million Taiwanese citizens and represented the real-world data in Taiwan (18). Patients’ personal data were encrypted before the data were released for research purposes. The study protocol conforms to the ethical guidelines of the 1975 Declaration of Helsinki.

### Study Design and Participants

**Figure 1:**
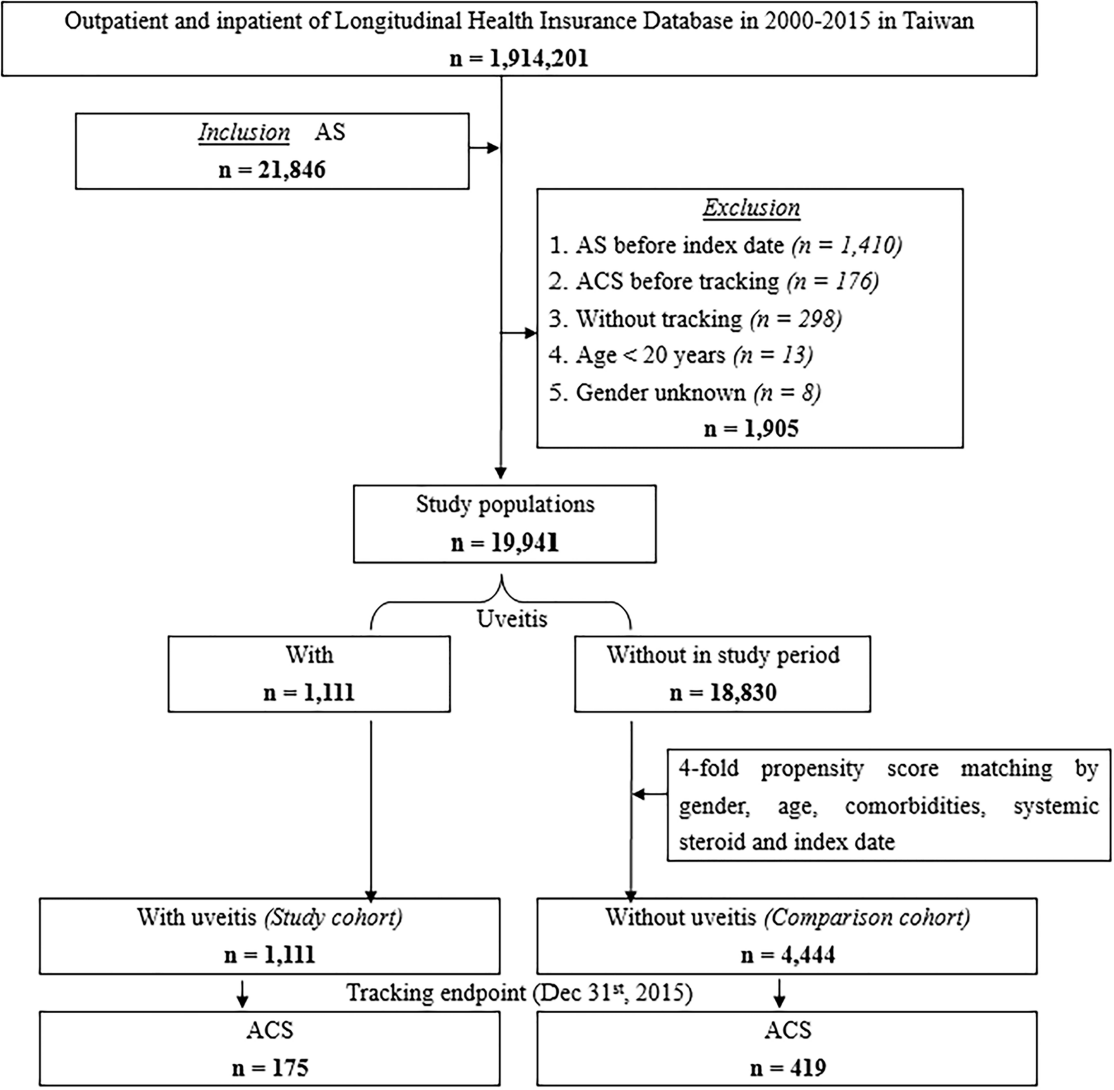
Flowchart of the study design.

We retrospectively conducted a matched-cohort study from January 1, 2000, to December 31, 2015. Patients were selected if they had at least one inpatient claim or more than three outpatient visits with the diagnosis of AS (ICD-9-CM code 720.0). The index date was defined as the date on which the patient was first diagnosed with AS. Patients diagnosed with AS or ACS before the index date were excluded to ensure that all ACS cases were newly diagnosed after AS. Patients aged 20 years and younger, of unknown sex, or without tracking follow-up were excluded. The study population was divided into uveitis (study cohort) and non-uveitis (comparison cohort) groups. The non-uveitis group was randomly matched fourfold with the uveitis group according to sex, age, and index year (under the same exclusion criteria). Furthermore, the uveitis group was divided into the anterior uveitis and posterior segment involvement groups. Patients were tracked until ACS onset or the end of the study period, whichever occurred first. The selection process is illustrated in **Figure 1**.

### Covariates

Uveitis was the main independent variable of interest. The evaluated covariates included sex, age, diabetes mellitus (DM), hyperlipidemia, hypertension (HTN), asthma, and chronic obstructive pulmonary disease (COPD). The considered medication was systemic steroids. These factors were matched at baseline and considered confounders and adjusted for statistical analysis.

### Statistical Analysis

All statistical analyses were performed using SPSS software version 22 (SPSS Inc., Chicago, IL, USA). The differences between the study participants and the comparison cohort for categorical and continuous variables were assessed using the chi-square and t-tests, respectively. A Cox proportional hazards model with Fine and Gray’s competing risk model was used to determine the hazard ratio of risk of ACS based on each variable. Survival analysis was performed using the Kaplan-Meier method with log-rank test. A two-tailed p value <0.05 was considered statistically significant.

### Ethics

This study was approved by the Institutional Review Board of the Tri-Service General Hospital (TSGHIRB: B-110-41), and the need for individual written informed consent was waived. This study was conducted in accordance with the code of ethics of the World Medical Association (Declaration of Helsinki).

## Results

As depicted in **Figure 1**, a total of 19,941 newly diagnosed patients with AS were identified, while 1,905 did not proceed past the exclusion criteria. Among them, 1,111 patients had uveitis (uveitis group), and a total of 18,830 patients had no uveitis during the study period; these patients were matched with a 4 fold-propensity score by sex, age, index date, and comorbidities. Then, 4,444 patients were included in the comparison cohort. These patients were followed up to ascertain the incidence of ACS.

**Table 1** shows the baseline characteristics and demographic characteristics of the study population. The mean age was 37.66 ± 19.10 years and 37.71 ± 19.91 years in study and comparison cohort, respectively. Men and women respectively accounted for 55.90% and 44.10% of the study population, while patients in the 20–39 age group constituted 59.68% of the study population. There were no significant differences in age, sex, comorbidities, and CCI-R between the study and comparison cohorts at baseline. At the study endpoint, ACS occurred in 175 (15.75%) patients with uveitis and in 419 (9.43%) patients without uveitis (p<0.001). The mean age at the endpoint was 40.68±20.20 years in the uveitis group and 41.48±20.53 years in the non-uveitis group, which was not significantly different (p=0.142). All-cause mortality was higher in the uveitis group (9.81%) than in the non-uveitis group (8.10%) (p=0.015). In **Table S1-1**, the mean follow-up time for the study and comparison cohort is 9.82 ± 8.40 years and 9.85 ± 8.55 years, respectively (p= 0.784). **Table S1-2** revealed that the average time in developing ACS in patients with AS is 3.01±3.21 years in the uveitis group and 3.75 ± 4.11 years in the non-uveitis group (p<0.001).

**Table 1:** Characteristics of the study and comparison population at baseline.

		Uveitis						
		Total		With		Without		*P*
		n	%	n	%	n	%	
**Total**	5,555		1,111	20	4,444	80	
**Gender**							0.999
Male	3,105	55.9	621	55.9	2,484	55.9	
Female	2,450	44.1	490	44.1	1,960	44.1	
**Age (years)**	37.70 ± 19.75		37.66 ± 19.10		37.71 ± 19.91		0.91
**Age group (yrs)**							0.999
20-39	3,315	59.68	663	59.68	2,652	59.68	
40-59	1,560	28.08	312	28.08	1,248	28.08	
≧60	680	12.24	136	12.24	544	12.24	
**Comorbidities**							
DM	1,398	25.17	275	24.75	1,123	25.27	0.689
Hyperlipidemia	317	5.71	63	5.67	254	5.72	0.911
HTN	1,143	20.58	230	20.7	913	20.54	0.892
COPD	879	15.82	176	15.84	703	15.82	0.952
Asthma	1,043	18.78	208	18.72	835	18.79	0.919
**Medications**							
Systemic Steroids	1,016	18.29	205	18.45	811	18.25	0.900
**CCI_R**			0.96 ± 1.12		0.92 ± 1.11		0.284

P, Chi-square/Fisher exact test on category variables and t-test on continue variables.

**Figure 2:**
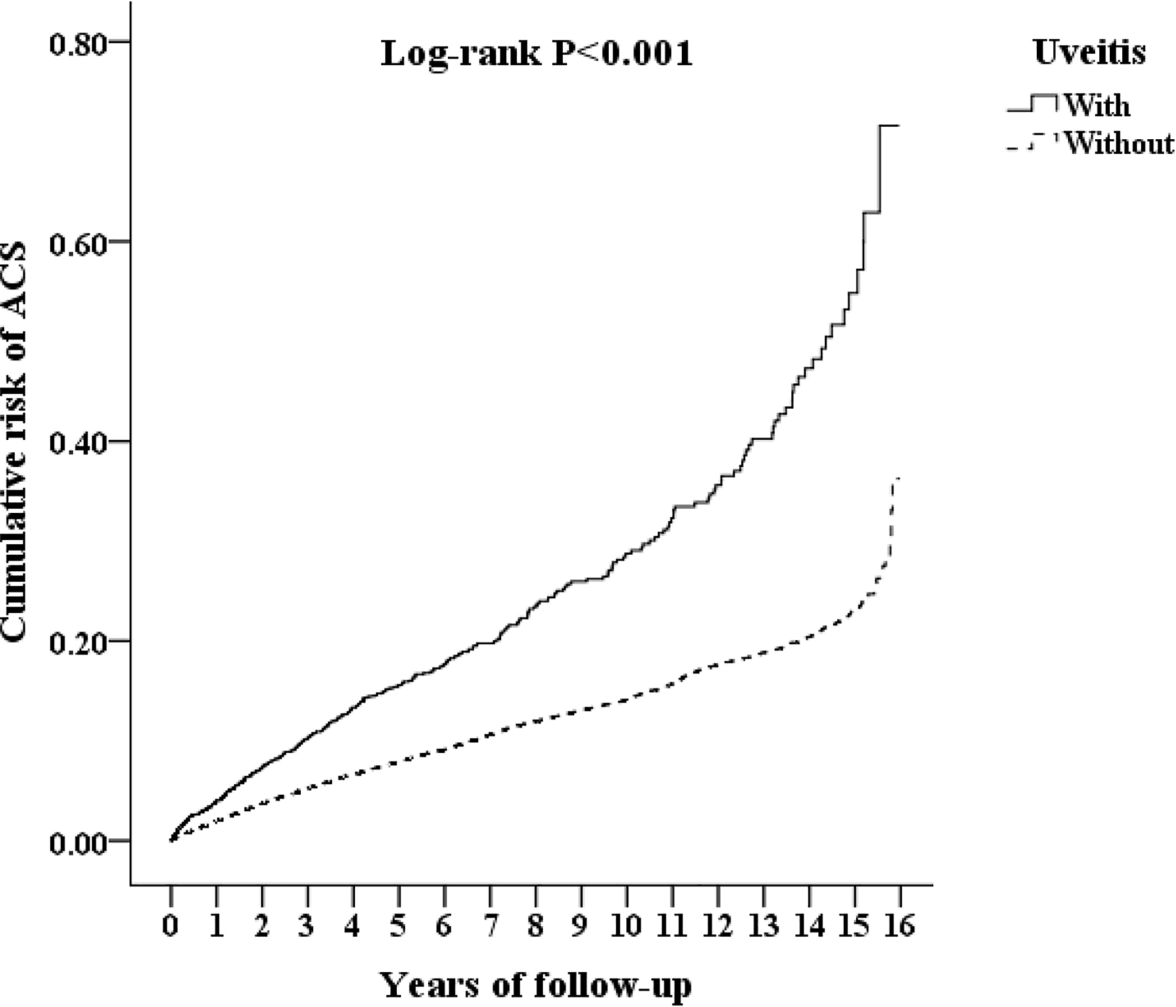
Kaplan-Meier curves for acute coronary syndrome (ACS) in patients with ankylosing spondylitis (AS), corresponding to the uveitis and non-uveitis groups. Line represents the uveitis group and dotted line represents the non-uveitis group.

**Figure 2** shows the cumulative risk of ACS in patients with AS with and without uveitis using the Kaplan-Meier method. The result demonstrates that uveitis group had a significantly higher risk of ACS than the non-uveitis group (log-rank test, P <0.001). The results of the Cox proportional hazards regression analysis using Fine and Gray’s competing risk model are shown in **Table 2**. The adjusted HR (aHR) for ACS in patients with AS and uveitis was 1.64 times that in those without uveitis (p<0.001). Furthermore, AS patients aged 40–59 years and ≥ 60 years had an increased risk for ACS (aHR=1.105 and 1.709, respectively). Surprisingly, no gender preference for ACS development was found. The aHR for DM, hyperlipidemia, HTN, COPD, and asthma were 3.080, 2.931, 3.336, 1.505, and 1.929, respectively. The aHR for CCI_R was 1.197 (P <0.001). Patients using systemic steroids had a higher risk of developing ACS (aHR: 2.017, p<0.001). This result demonstrates a higher risk of ACS in patients with AS with comorbidities than in those without these comorbidities.

**Table 2:** Factors of ACS by using Cox regression with Fine & Gray’s competing risk model.

	Crude HR (95%CI)	*P*	Adjusted HR (95% CI)	*P*
**Uveitis**	1.9 (1.628-2.055)	<0.001*	1.64 (1.470-1.906)	<0.001*
Female	Reference		Reference	
Male	1.202 (0.850-1.713)	0.192	1.173 (0.812-1.698)	0.236
**Age (yrs)**				
20-39	Reference		Reference	
40-59	1.114 (1.050-1.393)	0.017*	1.105 (1.011-1.340)	0.041*
≧60	1.727 (1.216-1.874)	<0.001*	1.709 (1.213-1.857)	<0.001*
**Comorbidities**				
DM	3.138 (2.362-5.345)	<0.001*	3.08 (2.157-5.247)	<0.001*
Hyperlipidemia	2.937 (1.906-4.963)	<0.001*	2.931 (1.807-4.879)	<0.001*
HTN	3.415 (2.479-5.754)	<0.001*	3.336 (2.285-5.451)	<0.001*
COPD	1.544 (1.907-2.581)	<0.001*	1.505 (1.062-2.504)	0.001*
Asthma	1.997 (1.137-2.738)	<0.001*	1.929 (1.114-2.714)	<0.001*
**Medication**				
Systemic Steroids	2.048 (1.065-2.998)	<0.001*	2.017(1.049-2.953)	<0.001*
**CCI_R**	1.215 (1.151-1.240)	<0.001*	1.197 (1.129-1.206)	<0.001*

HR, hazard ratio; CI , confidence interval; Adjusted HR: Adjusted variables listed in the table; Competing risk: All-caused mortality*denotes statistically significant.

The results of the stratified analyses of the risk factors associated with the development of ACS are shown in **Table 3**. The incidence of ACS was 1,593.38 per 100,000 person-years in the uveitis group and 953.40 per 100,000 person-years in the non-uveitis group. After adjustment for age, sex, and comorbidities, patients with AS and uveitis demonstrated an increased risk of ACS compared to those without uveitis (aHR: 1.675, p<0.001). The aHRs for ACS were 1.736 for men and 1.605 for women. Patients with AS and uveitis had an increased risk of ACS regardless of sex. A trend of increasing aHR for ACS was observed in patients with AS and uveitis as patient age increased (20–39 years, aHR: 1.390; 40–59 years, aHR: 1.578; ≥60 years, aHR: 4.188). This might indicate that age is a significant risk factor for ACS in AS patients with uveitis. Compared with the comparison cohort, the study cohort had a greater aHR for ACS in the absence or presence of comorbidities and systemic steroids (DM, 1.548, 3.091; hyperlipidemia, 1.581, 3.017; HTN, 1.52, 3.649; COPD, 1.653, 2.362; asthma, 1.69, 1.829; and systemic steroids, 1.510, 2.035, respectively). In addition, the presence of comorbidities increased the aHR for ACS.

**Table 3:** Factors of ACS stratified by variables listed using Cox regression with Fine & Gray’s competing risk model.

		Uveitis							
		With			Without				
		Events	PYs	Rate (per 10^5^ PYs)	Events	PYs	Rate (per 10^5^ PYs)	Adjusted HR (95% CI)	P
**Total**		175	10982.93	1593.4	419	43948.02	953.4	1.675 (1.501-1.947)	<0.001*
**Gender**									
**Male**		96	6135.11	1564.8	226	24511.12	922.03	1.736 (1.556-2.018)	<0.001*
**Female**		79	4847.82	1629.6	193	19436.9	992.96	1.605 (1.439-1.867)	<0.001*
**Age (yrs)**									
20-39		92	6135.2	1499.5	268	24563.12	1091.1	1.39 (1.245-1.615)	<0.001*
40-59		47	3076.11	1527.9	121	12508.97	967.31	1.578 (1.414-1.835)	<0.001*
≧60		36	1771.62	2032	30	6875.93	436.3	4.188 (3.752-4.869)	<0.001*
**Comorbidities**									
**DM**									
Without		134	8205.27	1633.1	383	35929.77	1066	1.548 (1.388-1.801)	<0.001*
With		41	2777.66	1476.1	36	8018.25	448.98	3.091 (2.768-3.591)	<0.001*
**Hyperlipidemia**									
Without		155	10346.68	1498.1	398	42112.88	945.08	1.581 (1.417-1.838)	<0.001*
With		20	636.25	3143.4	21	1835.14	1144.3	3.017 (2.702-3.506)	<0.001*
**HTN**									
Without		138	8715.83	1583.3	393	37258.89	1054.8	1.52 (1.362-1.767)	<0.001*
With		37	2267.1	1632	26	6689.13	388.69	3.649 (3.271-4.244)	<0.001*
**COPD**									
Without		158	9190.8	1719.1	395	37949.78	1040.9	1.653 (1.480-1.921)	<0.001*
With		17	1792.13	948.59	24	5998.24	400.12	2.362 (2.117-2.746)	<0.001*
**Asthma**									
Without		155	8900.8	1741.4	386	37080.38	1041	1.69 (1.515-1.965)	<0.001*
With		20	2082.13	960.55	33	6867.64	480.51	1.829 (1.638-2.126)	<0.001*
**Medications**									
**Systemic Steroids**									
Without	131		8846.9	1480.74	336	35462.77	947.47	1.51 (1.354-1.755)	<0.001*
With	44		2136.03	2059.90	83	8485.25	978.17	2.035 (1.825-2.365)	<0.001*

PYs, Person-years; Adjusted HR, Adjusted Hazard ratio: Adjusted for the variables listed in **Table 3**; CI, confidence interval; Competing risk: All-caused mortality*denotes statistically significant.

The uveitis group (n=1,111) was further grouped into anterior uveitis (n=394) and posterior uveitis (n=717) groups, as shown in **Table 4**. The overall incidence of ACS reported was 1,445.38 per 100,000 person-years in the anterior uveitis group and 1,676.30 per 100,000 person-years in the posterior uveitis group. The aHRs were 1.665 and 4.893 in the anterior uveitis and posterior uveitis groups, respectively.

**Table 4:** Factors of ACS among different uveitis subgroup using Cox regression with Fine & Gray’s competing risk model.

Uveitis subgroup	Populations	Events	PYs	Rate (per 10^5^ PYs)	Adjusted HR (95% CI)	P
**Without uveitis**	4444	419	43948.02	953.399038	Reference	
**With uveitis**	1111	175	10982.93	1593.38173	1.675 (1.501-1.947)	<0.001*
**Anterior uveitis**	394	57	3943.61	1445.37619	1.665 (1.495-1.935)	<0.001*
**Posterior segment involvement**	717	118	7039.32	1676.29828	4.893 (4.385-5.689)	<0.001*

PYs = Person-years; Adjusted HR, Adjusted Hazard ratio; Adjusted for the variables listed in **Table 3**; CI, confidence interval; Competing risk: All-caused mortality*denotes statistically significant.

## Discussion

In this 15-year population-based investigation on the correlation between development of ACS and uveitis in patients with AS, we found that patients with AS and uveitis showed a significantly increased risk of ACS compared to those without uveitis, even after adjusting for age, sex, and comorbidities. Similarly, Kaplan-Meier analysis revealed that the cumulative risk of ACS was significantly higher in the uveitis group than that in the non-uveitis group. Uveitis demonstrated a strong association with an increased risk of ACS in both sexes, all ages, and patients with AS with and without DM, hyperlipidemia, HTN, COPD, and asthma. In the subgroups of uveitis, both anterior uveitis and posterior segment involvement demonstrated an increased risk of ACS in patients with AS. This study identified uveitis as a risk factor for ACS in patients with AS and suggests that it may be a potential predictor of ACS in patients with AS.

The prevalence of AS ranges from 9 to 30 per 10,000 people in the general population (3, 19). Although the prevalence may vary from country to country, a male predominance is almost consistently observed (20). The male-to-female ratio was 2.79 in Taiwan and 3.8 in the US (21, 22). Our study population included patients with AS and uveitis, which showed a male-to-female ratio of 1.27, with a male prevalence of 55.90%. In general, the age of onset of AS is usually in the third decade and is consistent with the above studies (1, 2, 19); in this study, 59.67% of patients were aged 20–39 years.

Patients with AS tend to have a higher rate of comorbidities than the general population. The prevalence of HTN, DM, and asthma in patients with AS was 30.7%, 9.8%, and 2.2%, respectively, in the US (23), and the prevalence of HTN, DM and COPD was 25.7%, 7.1%, and 2.71%, respectively, in Spain (24). A systematic review and meta-analysis reported that the pooled prevalence of HTN, DM, hyperlipidemia, asthma, and COPD was 22.8%, 6%, 16.8%, 4.9%, and 1.8%, respectively (25). The prevalence of HTN, DM, hyperlipidemia, asthma, and COPD in our study population was 20.58%, 25.17%, 5.71%, 18.78%, and 15.82%, respectively. The observed differences may be attributed to the study design, environment, and ethnicity. In fact, prevalence of diabetes varies among different ethnicity, which may be because of environmental, lifestyle and genetic factors, and Asian populations have shown to have higher prevalence of diabetes than white populations (26). Since the comorbidities in our study were matched at baseline, the higher cumulative risk of ACS in the uveitis group found in this study was less likely to be caused by the baseline systemic diseases.

Aging, hypertension, and diabetes mellitus are associated with the development of ACS (27, 28) and are also risk factors for ACS in patients with AS. A nationwide study in Taiwan found that patients with AS with hypertension and diabetes had an aHR of 4.36 for ACS compared to patients without these comorbidities (6). A population-based study of the Swedish National Patient Registry compared the mortality of patients with AS with that of the general population and reported a hazard ratio of 1.60 with cardiovascular disease being the major cause of death (29). Furthermore, Backland et al. found that crude mortality in patients with AS was 14.5%, and 40% of cases were attributed to circulatory disease (30). Undeniably, patients with AS are at risk of ACS, and cardiovascular risk factors can only partially explain this excess cardiovascular risk. While the above-mentioned studies are findings for the general AS population, our study population included patients with AS and uveitis and found uveitis to be an independent risk factor for ACS.

Uveitis is the most common extraarticular manifestation of AS (31).The mechanism underlying the relationship between uveitis and ACS is unclear. Elevated interleukin (IL)-6, IL-8, and IL-17A in serum samples of active uveitis suggest that uveitis may be an indicator of disease activity and systemic inflammation in AS (32, 33). Inflammation is also involved in the entire process of atherosclerosis and destabilization of the plaque, ultimately leading to myocardial infarction (8, 34). The cytokines involved in the development of ACS include IL-1, IL-6, tumor necrosis factor alpha)-α, adipokines, chemokines, and interferons (35). Chronic inflammation can cause detrimental effects on endothelial cells in these coronary artery vessels (36). The association between uveitis and ACS may be due to similarities in the shared mechanisms of inflammation. This study reported a higher incidence of ACS in patients with AS and uveitis than in those without uveitis, indicating that uveitis is a new potential risk factor for ACS in patients with AS (aHR= 1.640, p<0.001).

In addition to inflammation, AS has a strong genetic component (37), and HLA-B27 remains the most highly associated gene (2). In addition, genome-wide association studies have found that genes encoding endoplasmic reticulum aminopeptidase (ERAP) and IL-23 receptors are involved in AS. ERAP-1 is involved in the preparation of peptides for binding to HLA class 1 molecules, which are later presented to immune effector cells. The IL-23/IL-17 axis is where IL-23 activates T-helper cells that facilitate the expansion and differentiation of Th17 cells to produce major pro-inflammatory cytokines (2, 38). In animal models, activation of the IL-23/IL-17 pathway and expansion of Th-17 cells contribute to spondyloarthritis-associated uveitis (39). Studies have also suggested an association between the IL-23/IL-17 axis and atherosclerosis (40). Patients with atherosclerosis have significantly increased levels of IL-23 in the plasma, and IL-23 levels are higher in carotid plaques than in nonatherosclerotic vessels (40). IL-17A blockade in apoE-deficient mice shows a reduction in atherosclerosis development and decreases the vulnerability of plaque (41), which suggests a similar pathway of ACS and uveitis in patients with AS.

Our study reported a higher aHR for ACS in the posterior segment involvement group than in the anterior uveitis group (**Table 4**). Noninfectious uveitis is the major subtype of uveitis in Taiwan (42). While anterior uveitis in spondyloarthropathies has been studied thoroughly, posterior segment manifestations have often been overlooked. However, Rodriguez et al. found that 24% of 29 seronegative spondyloarthropathy patients had retinal vasculitis, 94% had severe vitritis, and 76% had papillitis (43), indicating the importance of posterior segment manifestation in any spondyloarthropathy disease. Very few studies have examined cytokine levels in the different subtypes of uveitis. Using proteomics, Velez et al. analyzed vitreous samples from 15 patients with posterior uveitis, and among them, one AS patient showed upregulation of IL-23 and IL-17R (44). Uveitis and acute coronary syndrome may share a similar IL-23/IL-17 pathway. From the above studies, the posterior uveitis group may have more severe disease inflammation because of the elevation of IL-23 levels; however, further studies are needed to confirm this association. Nonetheless, both anterior uveitis and posterior uveitis were associated with an increased risk of ACS among patients with AS.

Clinically, the treatment of non-infectious uveitis (NIU) included topical steroids or systemic steroids, disease-modifying antirheumatic drugs (DMARDs, such as azathioprine, methotrexate, mycophenolate mofetil, cyclosporine, tacrolimus, cyclophosphamide, or chlorambucil), and biologic agents (such as adalimumab, or Infliximab) (45–47). Uveitis in AS patients belonged to one of NIU. In these patients, topical steroids are the major treatment for acute anterior uveitis. Nevertheless, uveitis with posterior or bilateral involvement, or refractory to topical medication, required systemic treatment such as systemic steroids, DMARDs, or biologic agents (45, 48). Under these conditions, systemic steroids were the first choice of treatment. However, high doses or long-term systemic steroids could cause serious systemic side-effects such as Cushing’s syndrome, hypertension, diabetic mellitus, hyperlipidemia, osteoporosis, …et al. The side effects of systemic steroids such as hypertension and hyperlipidemia could predispose treated patients to cardiovascular disease (49). Consequently, DMARDs or biologic agents were used to control inflammation and spare steroids in high-doses or prolong-used patients (45–48). Consistent with the literature (49), our study also found that using systemic steroids increased the risk of ACS in AS patients with uveitis. Nonetheless, the association of ACS between steroids and AS patients with uveitis still needs to be further investigated.

A strength of this study is the large sample size within a comprehensive database spanning over a 15-year period. This not only provides high statistical power that can best reflect real-world situations, but also minimizes selection bias. In addition, this is the first population-based study to investigate uveitis as a risk factor for acute coronary syndrome in patients with AS. Furthermore, this study matched comorbidities at baseline, which could provide more reliable results for adjusting these variables.

This study had several limitations that should be addressed. First, misclassifications of the subtype of uveitis and cardiovascular outcomes may be inevitable; however, the National Health Administration in Taiwan checks charts to ensure that patients have appropriate claims and treatment. Second, the LHID only included the population of Taiwan, thus, this result needed to be validated and extrapolated by a further study in a much bigger group. Third, due to that our research was a retrospective study by analyzing the fully anonymized database, we could not obtain other confounding variables (such as dietary habits, physical activity, smoking habits, environmental factors, regional difference, body mass index, and laboratory data). Hence, the interferences of these confounding variables needed a further prospective study to determine these.

In conclusion, this study demonstrated that uveitis is an independent risk factor for ACS in patients with AS. In patients with AS and uveitis, a trend of specific comorbidities showed a higher risk of ACS. The results of this study will allow ophthalmologists, rheumatologists, or any medical doctor to be aware of the risk of ACS when monitoring patients with AS and uveitis.

## Data Availability Statement

The original contributions presented in the study are included in the article/**Supplementary Material**. Further inquiries can be directed to the corresponding author.

## Ethics Statement

The studies involving human participants were reviewed and approved by Institutional Review Board of the Tri-Service General Hospital (TSGHIRB: B-110-41). Written informed consent for participation was not required for this study in accordance with the national legislation and the institutional requirements.

## Author Contributions

KF, W-CC, Y-HC, J-TC, and C-LC: study design and manuscript writing. W-CC, C-AS, C-HC, and C-LC: data extracting and statistical analysis. K-MF, Y-HC, C-AS, J-TC, and C-LC: data checking. All authors contributed to the article and approved submitted version.

## Funding

This study was funded by the Tri-Service General Hospital Research Foundation (TSGH-D-110112 and VTA111-V1-1-2) and by the Ministry of Science and Technology (MOST 107-2314-B-016-030). The sponsors have no role in study design, data collection and analysis, decision to publish or preparation of the manuscript.

## Conflict of Interest

The authors declare that the research was conducted in the absence of any commercial or financial relationships that could be construed as a potential conflict of interest.

## Publisher’s Note

All claims expressed in this article are solely those of the authors and do not necessarily represent those of their affiliated organizations, or those of the publisher, the editors and the reviewers. Any product that may be evaluated in this article, or claim that may be made by its manufacturer, is not guaranteed or endorsed by the publisher.
