# Supplementary material for: Increased Risk of Acute Coronary Syndrome in Ankylosing Spondylitis Patients With Uveitis: A Population-Based Cohort Study
Source: Front Immunol. 2022 Jun 10;13:890543. doi: 10.3389/fimmu.2022.890543 (PMC9226308; doi:10.3389/fimmu.2022.890543)
Supplement: Supplementary file 1 [file Table_1.docx]

Supplementary Material

# Supplementary Tables

Table S1-1. The years of follow-up for study population

| **Uveitis** | **Min** | **Median** | **Max** | **Mean ± SD** | ***P*** |
| --- | --- | --- | --- | --- | --- |
| Total | 0.01 | 7.77 | 15.99 | 9.86 ± 8.52 | 0.784 |
| With | 0.01 | 7.68 | 15.99 | 9.82 ± 8.40 |  |
| Without | 0.01 | 7.78 | 15.99 | 9.85 ± 8.55 |  |

Table S1-2. The years to acute coronary syndrome (ACS) in study population

| **Uveitis** | **Min** | **Median** | **Max** | **Mean ± SD** | ***P*** |
| --- | --- | --- | --- | --- | --- |
| Total | 0.03 | 2.32 | 15.89 | 3.59 ± 3.98 | <0.001 |
| With | 0.03 | 2.03 | 15.81 | 3.01 ± 3.21 |  |
| Without | 0.03 | 2.58 | 15.89 | 3.75 ± 4.13 |  |
